# Very Late Bare Metal Stent Thrombosis

**DOI:** 10.1155/2013/856095

**Published:** 2013-08-21

**Authors:** Mariana Soto Herrera, José A. Restrepo, Andrés Felipe Buitrago, Mabel Gómez Mejía, Jesús H. Díaz

**Affiliations:** ^1^Critical Care Department, Hospital Universitario Fundación Santa Fe de Bogotá, Calle 119, No. 7-75, Bogotá 110111, Colombia; ^2^Universidad de los Andes, Carrera 1, No. 18A-12, Bogotá 111711, Colombia; ^3^Internal Medicine Department, Cardiology Section, Hospital Universitario Fundación Santa Fe de Bogotá, Calle 119, No. 7-75, Bogotá 110111, Colombia

## Abstract

Very late stent thrombosis is a rare and not-well-understood complication after bare metal stent implantation. It usually presents as an ST elevation acute coronary syndrome and it is associated with high rates of morbidity and mortality. Pathophysiologic mechanisms are not well defined; nevertheless, recent studies have proposed a neoatherosclerotic process as the triggering mechanism. We present the case of a patient with bare metal very late stent thrombosis 12 years after implantation.

## 1. Case Presentation

**Figure 1:**
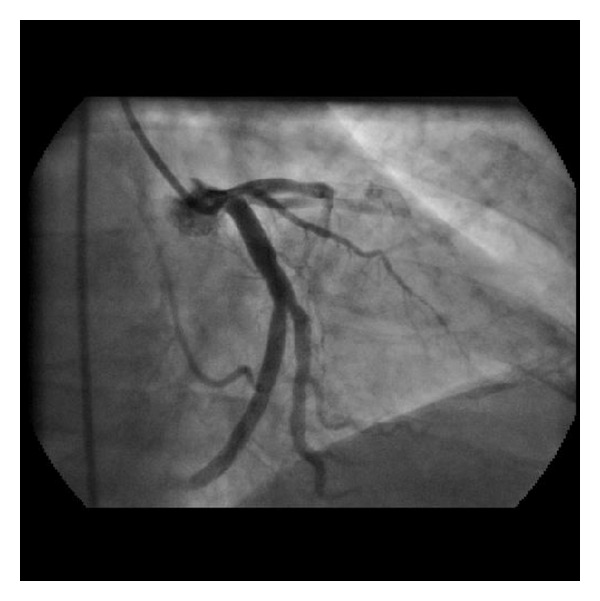
Total thrombotic occlusion of the previously stented segment of the LDA. LDA: left descending artery.

**Figure 2:**
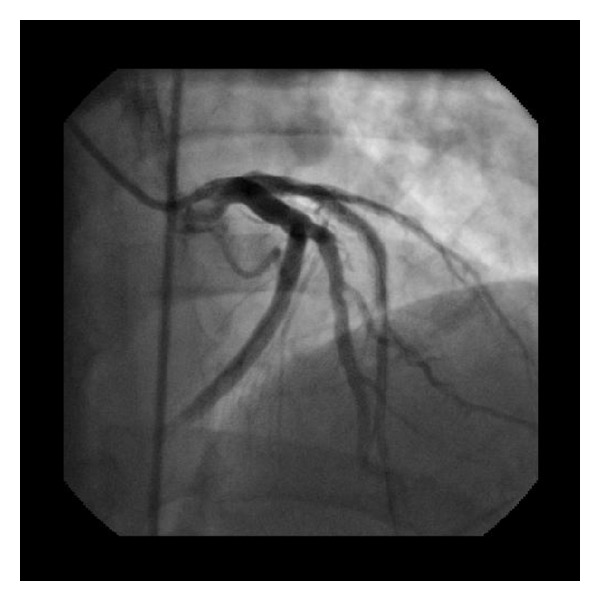
Restoration of perfusion of  LDA to distal TIMI 3 flow.

In 2000, a 66-year-old man was admitted to the emergency department (ED) for acute retrosternal chest pain, intensity 10/10, irradiated to neck and associated with dyspnea and diaphoresis; within the last week he had had similar episodes. Patient was an active smoker. In the ED an electrocardiogram was performed, showing acute changes suggesting anterior wall myocardial infarction. Thrombolytic therapy with streptokinase was used and the patient was derived to the catheterization laboratory. Coronary angiography revealed subtotal occlusion of the proximal and middle left anterior descending coronary artery (LDA), balloon angioplasty with a 3 × 20 mm balloon was performed at 8 atm, and two bare metal stents were implanted: ML 3.5 × 18 mm and ML 3.5 × 23 mm respectively, with excellent angiographic results. Patient was discharged with aspirin, warfarin, diuretics, and ACE inhibitor. In September 2012 patient was admitted to the ED for intermittent acute chest pain. Twelve lead electrocardiogram evidenced anterior ST segment elevation. After administration of aspirin 300 mg and ticagrelor 180 mg, patient was driven to the catheterization laboratory. Coronary angiography revealed total thrombotic occlusion of the previous stented segment of the LDA with an abrupt cutoff of septal and diagonal branches (Figure 1). Predilatation with a Splinter balloon 2.5 × 21 mm was performed at 18 atm and stent Endeavour Resolute Integrity 3.8 × 38 m was implanted at 18 atm, restoring perfusion to distal TIMI 3 flow (Figure 2). Patient had an adequate clinical course and was discharged with dual antiplatelet treatment with aspirin and ticagrelor, beta blocker, and statin. 

## 2. Discussion

Coronary percutaneous intervention with stenting is one of the most widely performed procedures for the treatment of symptomatic coronary artery disease. Even though drug eluting stents have diminished bare metal stent limitations, there remain serious concerns about late complications as intra stent restenosis and very late stent thrombosis (VLST) [1]. VLTS is an exceeding rare phenomenon with bare metal stents, and it occurs most often with drug eluting stents. Recent studies have demonstrated an annual incidence of approximately 0.5% in the former that reaches up to 2% in the latter. Most cases present as an ST segment elevation acute coronary syndrome carrying high morbidity and reaching annual mortality rates of  10% to 20% [2]. 

In order to settle unified definitions, the Academic Research Consortium developed a precise definition of stent thrombosis depending on event certainty and time frame. Diagnosis of definite stent thrombosis is made only after confirmation by coronary angiography and autopsy if the patient died. Probable stent thrombosis is considered when acute myocardial infarction diagnosis is made in the territory of the previously stented artery. Possible stent thrombosis is considered when there is an unexplained death within 30 days of stent implantation. Time frame occurrence is divided into early (30 days), late (within 12 months), or very late (after 1 year) [3, 4]. 

There is a lack of certainty regarding the pathophysiologic mechanisms of this complication. In the case of bare metal stents which have a rapid reepithelization, the inflammatory response does not seem to be the triggering cause. Recent studies have proposed a neoatherosclerotic process and the subsequent instability and plaque rupture as the triggering mechanism of this complication [1, 5]. Histological comparisons of thrombi in patients with VLST versus patients with acute coronary syndrome unrelated to stent thrombosis show similar findings, mainly, atherosclerotic plaques made of foamy macrophages, cholesterol crystals, and a thin fibrous cap [5]. 

We report a case of very late stent thrombosis occurring 12 years after stent implantation. According to the literature review, this is the second case with the widest period of time of stent thrombosis since stent implantation, preceded by a case report of 13 years. Nevertheless uncertainty remains whether the acute coronary syndrome was indeed due to very late stent thrombosis or rather due to a new emerging pathology: neoatherosclerosis as the overlying mechanism in cases with such a long time between stent implantation and thrombosis occlusion.
